# Supplementary material for: Oral Lisinopril Raises Tissue Levels of ACE2, the SARS-CoV-2 Receptor, in Healthy Male and Female Mice
Source: Front Pharmacol. 2022 Mar 10;13:798349. doi: 10.3389/fphar.2022.798349 (PMC8961328; doi:10.3389/fphar.2022.798349)
Supplement: Supplementary file 2 [file DataSheet1.pdf]

Oral Lisinopril Raises Tissue Levels of ACE2, the SARS-CoV-2 Receptor, in Healthy Male and Female Mice

Approach

Experimental procedure:

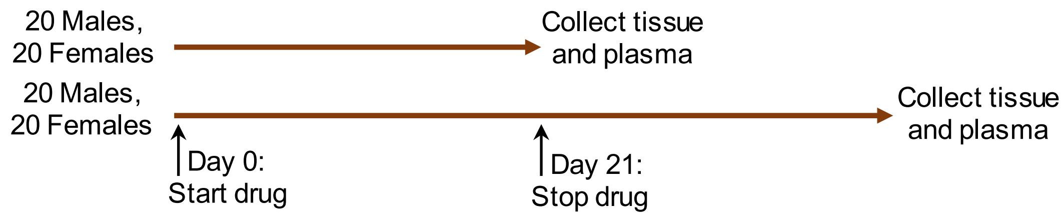

Drug treatment groups:

- Lisinopril
- Losartan
- Lisinopril + Losartan
- Vehicle control

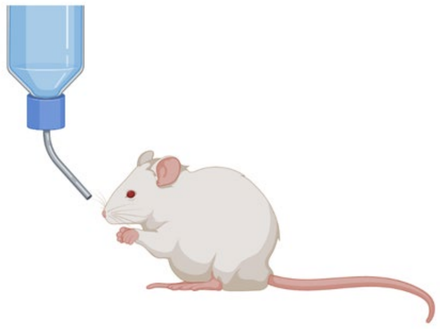

Tissues evaluated:

- Small intestine
- Lung
- Kidney
- Brain

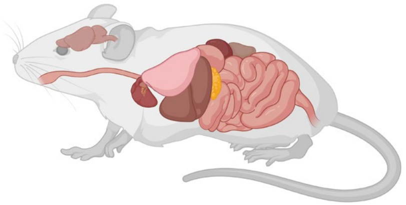

Primary Endpoint: $\text{ACE2 Protein Index} = \frac{[\text{Tissue ACE2}]}{[\text{Tissue protein}]}$

Primary Findings

Oral lisinopril increased ACE2 in tissues.

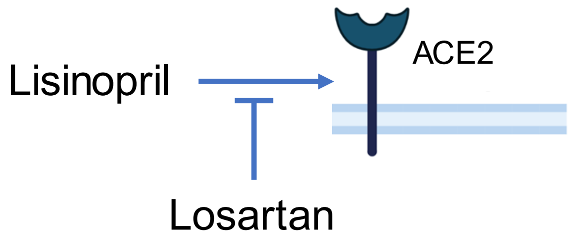

The addition of losartan prevented the increase in tissue ACE2.

Secondary Findings

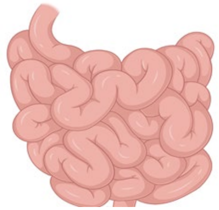

ACE2 index was highest in the small intestine.

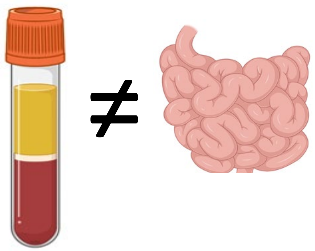

Plasma ACE2 did not correlate with ACE2 in any tissue, indicating it is not a good biomarker for tissue ACE2

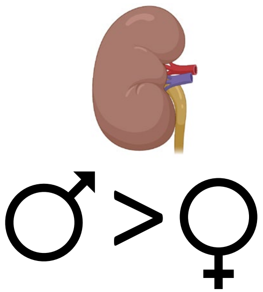

In the kidney, ACE2 index was greater in males than in females.

Brooks et al., 2022 ***Frontiers in Pharmacology***

https://www.frontiersin.org/articles/10.3389/fphar.2022.798349/full
